# The Polyol Process and the Synthesis of ζ Intermetallic Compound Ag_5_Sn_0.9_

**DOI:** 10.3390/ma15228276

**Published:** 2022-11-21

**Authors:** Roland Mahayri, Mohammed Ali Bousnina, Silvana Mercone, Ky-Lim Tan, Jean-Michel Morelle, Frédéric Schoenstein, Noureddine Jouini

**Affiliations:** 1 Laboratoire des Sciences des Procédés et des Matériaux (UPR3407), CNRS-USPN, 93430 Villetaneuse, France; 2 GREMAN UMR7347—CNRS, Université de Tours, Parc Grandmont, 37200 Tours, France; 3 Valeo Equipements Electriques Moteurs SAS, 2 rue André Boulle, BP 150, 94017 Créteil, France

**Keywords:** ζ-Ag_4_Sn submicrometric particles, polyol synthesis, microstructure, die-attach material

## Abstract

The present work concerns the intermetallic compound (IMC) existing in the Ag–Sn system and its potential use in electronics as attachment materials allowing the adhesion of the chip to the substrate forming the power module. First, we present the synthesis protocol in polyol medium of a compound with the chemical formula Ag_5_Sn_0.9_ belonging to the solid solution of composition located between 9 and 16 at.% Sn, known as solid solution ζ (or ζ-Ag_4_Sn). This phase corresponds to the peritectic invariant point at 724 °C. Differential thermal analysis and X-ray dispersive analysis confirm the single-phased (monocrystalline) nature of the Ag_5_Sn_0.9_ powder issued after synthesis. Scanning electron microscopy shows that Ag_5_Sn_0.9_ particles are spherical, and range in submicronic size of around 0.18 μm. X-ray diffraction analysis reveals that the ζ phase mostly exists under the two allotropic varieties (orthorhombic symmetry and hexagonal symmetry) with however a slight excess of the hexagonal variety (60% for the hexagonal variety and 40% for the orthorhombic variety). The lattice parameters resulting from this study for the two allotropic varieties are in good agreement with the Hume-Rothery rules.

## 1. Introduction

Historically, in power electronics, lead solders based mainly on Sn–Pb alloys were used as attachment joints because of their stable behavior [1]. To obtain the power module, a joint based on a lead alloy with a general eutectic composition is interposed between the various components to be assembled. The entire device is then brought to a temperature slightly above the melting point of the eutectic and then cooled slowly. This brazing process allows the diffusion of the elements of the alloy to the different components and leads to their assembly. However, the European Restriction of the use of certain Hazardous Substances in electrical and electric equipment (RoHS) directive dating from 2002 forbade the use of lead and certain harmful substances such as mercury, cadmium, etc.; and thus intended to gradually replace Sn–Pb alloys in current applications. In this context, great effort was deployed to propose alternative lead-free binary or ternary eutectic alloys, such as Sn–Zn [2,3], Sn–Sb [2,4], Sn–Ag [5,6,7] and SnAgCu [8,9]. However, all of these alloys have relatively low melting points (between 80 °C and 240 °C), which make them unsuitable for electronic power components intended for applications in extreme conditions (high temperature, etc.) such as electric aircraft for the aeronautical industry, the electrification of vehicles in the automotive, or the rail sector [3].

Faced with these limitations, a significant number of studies has been devoted to the development of new processes for assembling the components of power modules. In this context, several works have been devoted in recent decades to the use of silver as an attachment material, the assembly being carried out by means of the sintering process [10,11] instead of brazing. In comparison with the brazing process, silver sintering offers two advantages. On the one hand, it is an easy-to-implement process for assembling the power module (chip assembly/joint/substrate) without resorting to a liquid state step. The assembly is then achieved by solid-state atomic diffusion processes activated by the heat treatment. Unlike brazing, the composition of the attachment material is much more simplified. It is here a single element (Ag) whereas in the brazing process the attachment material is in most cases a mixture of phases or a compound formed of several elements (IMCs for example). On the other hand, the great interest shown for this alternative in the field of power electronics (packaging) is justified by the excellent properties of silver as an attachment material, i.e. its high melting point (961 °C), excellent electrical and thermal conductivities (63 × 10 S·m^−1^ and 429 W/m·K) and excellent resistance to oxidation and corrosion, combined with its good mechanical strength [12,13].

It should, however, be noted that due to its high melting point (961 °C), the temperature required for the sintering of the bulk material (0.8Tf ~ 714 °C) cannot be applied for the assembly of the power modules without deterioration of one of the components. To keep the power module components safe, sintering must be carried out at a temperature below 300 °C. This essentially depends on four parameters: the temperature, the pressure applied to the assembly, the time and the size of the silver particles, this last parameter playing an important role. Thus, particles of micrometric size (1–20 microns) can only be sintered if the pressure applied is relatively high (40 MPa) and at a temperature of 240 °C [14]. Such high pressure is a source of deterioration of the module and in particular the chip [15]. Conversely, the sintering of silver particles of nanometric size can be achieved by applying much lower pressures or even without pressure if the particle size is sufficiently reduced. This last process seems the most promising in the assembly of the components of the power module as it has the advantage of lower cost, ease of implementation and preserves the integrity of the module components [16,17,18].

Despite these advantages, this assembling process has two major drawbacks. First, the relatively high price of silver and secondly, because of their health risk, the use of nanoparticles. This work is part of a project aimed at replacing silver with less expensive compounds whose particles have a size beyond the nanometric range (greater than 100 nm) in order to prevent health risks. Our choice fell on the compounds existing in the Ag–Sn system, namely the intermetallic compound Ag_3_Sn and the solid solution Ag–Sn noted ζ and represented in the literature by the phase Ag_4_Sn and with compositions varying in the range of 9–16 at.% Sn as shown in the corresponding phase diagram [19].

Ag_3_Sn and ζ-phases can be good candidates in the sintering process for interconnection in electronic power devices. Indeed, these compounds have relatively high melting points of 480 °C and 724 °C, respectively, which offer a thermal stability at different operating temperatures, and, at the same time, their electric and thermal properties are close to those of the eutectic alloy Sn–3.5Ag as well as several lead-free alternatives frequently used in the market [1,20,21]. Additionally, when comparing their melting points with that of silver (914 °C), lower processing temperatures are more likely possible.

In a first article, the synthesis in polyol medium of submicron particles of Ag_3_Sn was described [22]. The polyol process is a soft chemistry synthesis method developed by F. Fiévet et al. in 1989 to prepare polymetallic spherical particles at micron (even submicron) sizes, generally with easily reducible metals such as copper, gold, palladium, silver and their alloys [23,24,25,26]. This was then extended to the synthesis of other classes of inorganic materials: oxides, hydroxides [27,28] and chalcogenides [29]. This process takes advantage of the reducing power of polyols. Easily reducible metals such as cobalt, nickel and iron were subsequently produced by this process [23]. In the polyol process it is possible to control the nucleation and growth steps. The α-diol type polyols, such as 1,2-ethanediol (ethylene glycol, EG), 1,2-propanediol, 1,2-butanediol, 1,5-pentanediol, etc., are the most widely used. Compounds resulting from intermolecular dehydration by condensation of α-diol, such as diethylene glycol (DEG or 2-hydroxyethyl ether) have also been used. The polyol maintains a metallic precursor in suspension (such as salts based on chloride, acetate, nitrates, hydroxides and oxides, etc.). By heating at moderate temperature we can favor reduction or forced hydrolysis resulting in the formation of metals, oxides or hydroxides via the nucleation and growth stages. The competition between the reduction and hydrolysis reactions is controlled by the hydrolysis rate, which is defined as the ratio of the number of moles of water to that of metal cation. The reduction reaction is favored when the hydrolysis rate is low or even zero.

The polyol solvent can also be a surfactant which is adsorbed on the surface of the particles to prevent their agglomeration by steric gene (as a stabilizer that limits particle growth and prevents agglomeration [23,30,31,32]). In addition, their high boiling point (of the solvents) allows them to solubilize a large number of metal salts and to activate reactions such as the oxidation of the polyol and consequently the formation of metals by reduction.

In the present work, the polyol process was extended to the elaboration of the solid solution and particularly the phase ζ with the chemical formula Ag_5_Sn_0.9_. In addition, it is possible to conduct these syntheses on a semi-pilot scale and thus have enough material for the die-bonding process. As will be shown hereafter, this phase has a very close structural filiation with the intermetallic compound Ag_3_Sn and therefore X-ray diffraction analysis does not allow its unambiguous characterization. During this study, thermal (DTA) as well as chemical (EDX) analyses were essential for a precise characterization of these phases. The results of these analyses are compared with the expected phases according to the equilibrium diagram and confirm that the obtained phase belongs to ζ- phases and corresponds to the chemical formula Ag_5_Sn_0.9_.

## 2. Experimental Procedure

### 2.1. Materials

The materials used in this study to synthesize the Ag_5_Sn_0.9_ particles were tin (II) chloride (SnCl_2_, 99%, Honeywell, Charlotte, NC, USA), silver nitrate (AgNO_3_, >99%) and ethylene glycol (EG) (VWR, 98%, Radnor, PA, USA) (Alfa Aesar, 99%, Haverhill, MA, USA). This latter is used as a solvent and a reducing agent for silver ions. Sodium borohydride (NaBH_4_) was selected as a reducing agent for tin ions. A surface stabilizer, PVP (Alfa Aesar), was used to prevent particles from coalescing. A distillation device [21] was chosen to achieve a lower hydrolysis rate and obtain the metallic particles. Note that all the chemical products were used as received, without any further purification.

### 2.2. Synthesis

The synthesis was carried out using a distillation assembly comprising a heating mantle, a temperature probe, a temperature regulator, a three-necked flask, a stirring shaft and a distillation column [21]. A volume of 200 mL of EG was poured into a 500 mL three-necked flask. The polyol was under an ambient atmosphere and underwent mechanical stirring at 350 revolutions per minute (rpm). Table 1 resumes the optimal conditions to obtain the Ag_5_Sn_0.9_ phase. At 50 °C, 1.43 g of PVP58000 were added and dissolved in the polyol medium. Subsequently, 1.43 g of AgNO_3_ were added at 70 °C to form a mass ratio of PVP/AgNO_3_ = 1. The solution became yellowish after dissolution of the precursors. The rate of temperature increase was set at 5 °C./min. After a plateau of 5 min at 160 °C, 1.4 g of SnCl_2_ were added to the polyol to constitute a molar ratio n (Ag/Sn) = 1.14. After an additional 5 min at 160 °C, stirring was stopped and a mass of 5.478 g of NaBH4 corresponding to a molar ratio of NaBH_4_/Sn = 19.6 was slowly added to the medium. Then, the solution was brought to 180 °C with a plateau of 1 h maintained at this temperature while it was stirred mechanically. At the end of the reaction, the solution was cooled by water quenching (the heating mantle was removed from the experimental device). The powder was then isolated by centrifugation at 12000 rpm for 10 min and washed with ethanol and acetone, alternately. These steps were necessary in order to remove the remains of the polyol. Washing was stopped when the float became colorless. Finally, the powder was dried in an oven at 70 °C for 12 h. It is interesting to note that the as-produced powder was in a metastable state in comparison with the predictions of the equilibrium diagram of the Ag–Sn system. 

### 2.3. Characterizations

The crystal structure of the synthesized powder was determined by x-ray diffraction (XRD) (20° < 2Ɵ <120° and Δ2Ɵ = 0.03°), using Co Kα1 radiation (Kα1 = 1.78897 Å) on an INEL Equinox 1000 X-ray diffractometer. XRD characterization was performed by using two softwares: Match3 (version 3.14, CRYSTAL IMPACT, Bonn, Germany) was used for the phase identification, and material analysis using diffraction (Maud) (version 2.993, L. Lutterotti, University of Trento, Trento, Italy) was used to perform the Rietveld refinement analysis. The background and incident intensity factors were refined first; lattice constants were refined in a second step. Additional characteristics such as texture, crystallite size, strain and atomic positions of silver and tin were refined in followed steps. When necessary, phase percentages are refined as well. In addition, phase identification was carried on by differential thermal analysis (DTA) using Labsys TG-D747 instrument. The morphological observations and the particle/grain size were achieved by scanning electron microscopy (SEM), using a field emission gun scanning electron microscopy (FEG-SEM) model ZEISS TM SUPRA 40 VP.

## 3. Results

### 3.1. DTA and EDX Analysis

**Figure 1:**
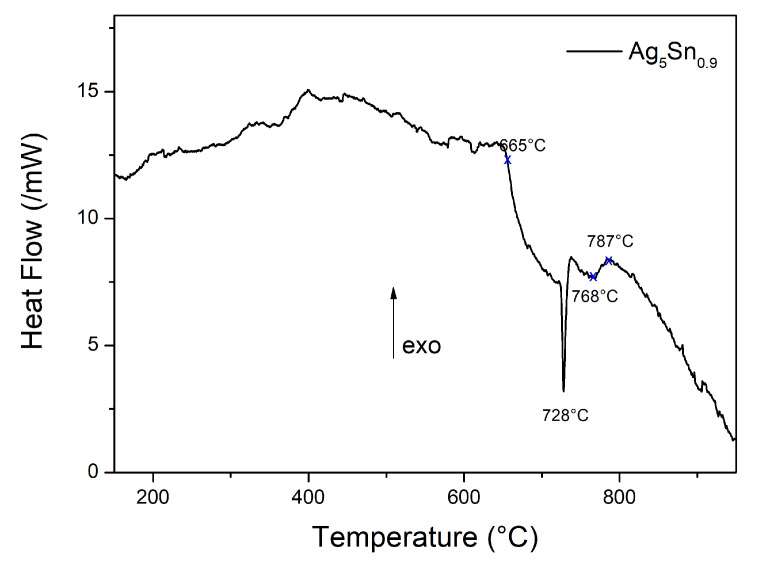
Thermogram of Ag_5_Sn_0.9_ particles.

The thermogram of Figure 1 indicates the formation of the ζ- phase which is characterized by the endothermic peak at around 728 °C, characteristic of the peritectic transformation (at 724 °C) due to the fusion of the ζ phase (Equation (1)):

ζ (15 at.% Sn) ⇌ Solid solution (11.5 at.% Sn) + Liquid (19.5 at.% Sn). (1)

**Figure 2:**
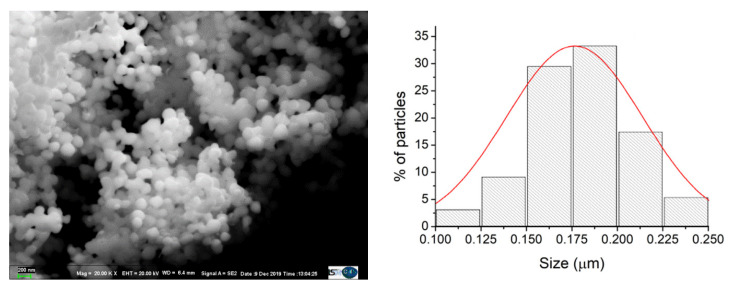
SEM photo and particle size distribution of Ag_5_Sn_0.9_ particles.

As shown in Figure 2, the particles obtained are spherical, submicronic and non-aggregated, with an average size equal to 175 nm.

**Figure 3:**
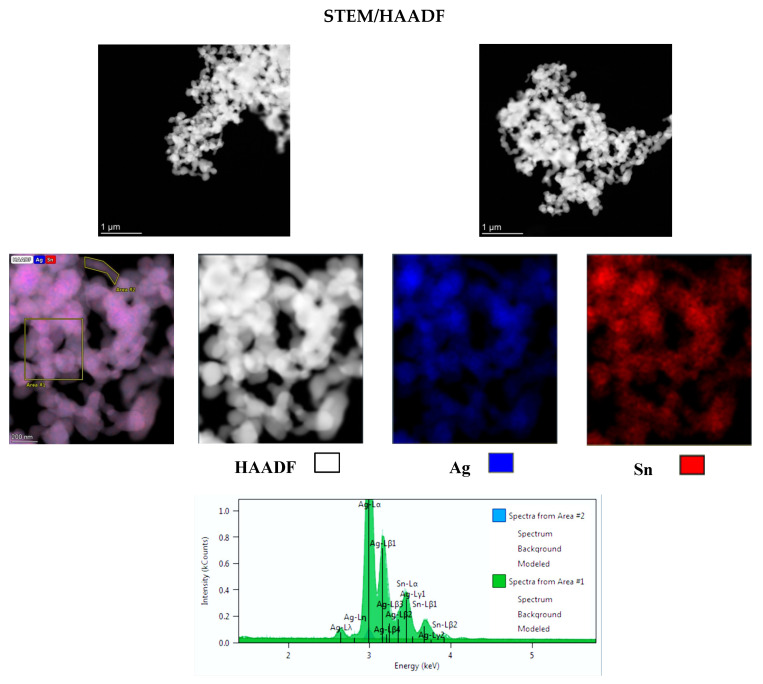
TEM images of Ag_5_Sn_0.9_ particles and elemental analysis.

The chemical composition of the ζ phase has been precisely determined using the scanning transmission electron microscopy/high-angle annular dark-field imaging technique (STEM/HAADF). Observations and analysis were carried out on two different areas (Figure 3).

Chemical imaging analysis revealed a homogeneous distribution of silver and tin in all the particles (Figure 3). The composition is, however, variable from one area to another but remains within a relatively narrow range close to 1.3% (Table 2). Indeed, for silver, the percentage varies between 84.90% and 86.22% and for tin it varies between 15.10% and 13.78%. Based on this, the average chemical composition for the ζ phase can be estimated at 85.56 at.% Ag and 14.46 at.% Sn. This chemical composition indicates the formation of a ζ phase with a chemical formula close to Ag_5_Sn (Ag_5_Sn_0.9_) and not Ag_4_Sn.

**Figure 4:**
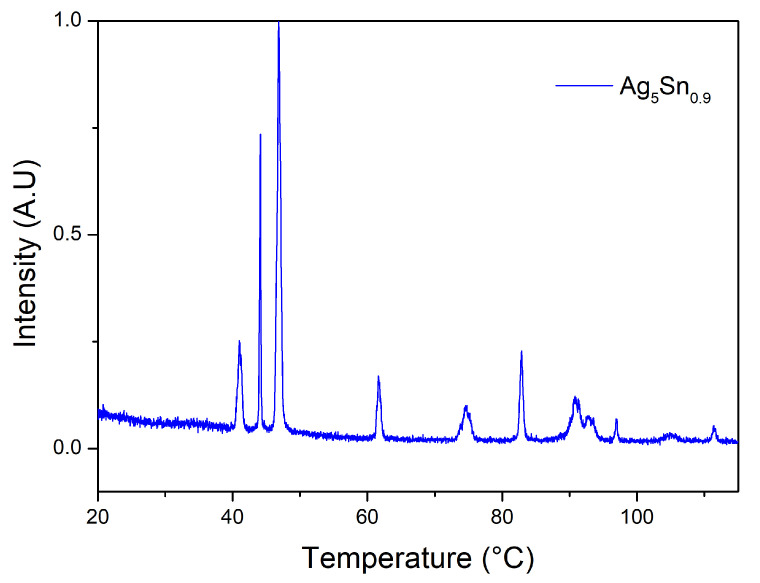
X-ray diffractogram of Ag_5_Sn _0.9_ particles.

Finally, we conducted XRD analysis on our sample powder. As a reminder, the DTA and EDX analyses show that the obtained phase corresponds to the ζ phase. As seen in Figure 4, and as discussed above as well as in the previous work on Ag_3_Sn [22], the diffractogram is substantially identical between the Ag_3_Sn and the ζ phases and cannot discriminate between them. This point will be taken up in the following discussion. The crystallite size calculated by MAUD (174 nm) is very close to the size of the particles deduced from SEM observations, indicating that the particles obtained are monocrystalline and probably formed by a mechanism involving Ostwald ripening.

### 3.2. Structural Hypothesis for the ζ Phase

As pointed out above, the characterization of the elaborated phase was carried out essentially on the basis of the DTA and EDX analysis based on the reading of the equilibrium diagram of the Ag–Sn system. Indeed, XRD analyses are not suitable for this purpose because of the similarity of the diffractograms of the two phases Ag_3_Sn and ζ-Ag_4_Sn (or Ag_5_Sn_0.9_). An attempt to explain this similarity is proposed in the following paragraphs.

#### 3.2.1. Review on the Structure of the Ag_3_Sn Phase

**Figure 5:**
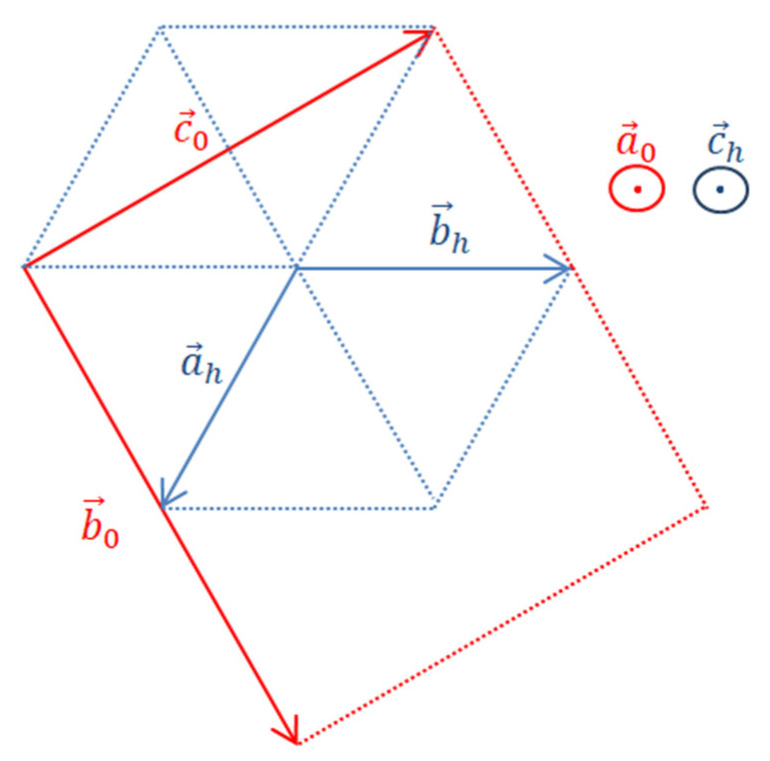
Diagram of correspondence between the two hexagonal and orthorhombic crystal systems.

The Ag_3_Sn phase exists in two allotropic varieties with a phase transition at 395 °C [33,34]. The high temperature variety has hexagonal symmetry (Space Group P6_3_/mmc). The low-temperature variety has orthorhombic symmetry (Space Group Pmmn) and results from a distortion of the hexagonal variety. Figure 5 shows the correspondence diagram between the two cell parameters, taking into account the permutation of the axes carried out by Rossi et al. [33]. In this case, the orthorhombic lattice parameters ($a_{0}$,$\;b_{0\;},\;c_{0})$ derive from those of the hexagonal lattice ($a_{h},$ $c_{h})$ by the following relations (Equation (2)):

(2) $$a_{0}=c_{h}\;;\;b_{0\;}\approx 2\;a_{h}\;;\;c_{0}\approx \;a_{h}\;\sqrt{3}$$

As noted above, Ag_3_Sn has been synthesized by the polyol process. The refinements of its X-ray patterns were more satisfactory when the hexagonal phase is taken into consideration. The low-temperature variety with orthorhombic symmetry was certainly the majority (89%) but the high-temperature (hexagonal) variety is still present in a non-negligible amount (11%) [22]. The presence of both varieties at room temperature is likely due to the nature of the polyol process. Indeed, it has been shown that this method belongs to the soft chemistry routes which enable the formation of metastable phases along with stable ones [35,36].

#### 3.2.2. Structural Hypothesis for the ζ Phase

The solid solution ranging from 9 to 16 at.% Sn (ζ) also has hexagonal symmetry and belongs to the same space group (P6_3_/mmc) as the high temperature variety of the Ag_3_Sn phase. In addition, the lattice parameters of the Ag_4_Sn phase representative of this family are close to those of the high temperature Ag_3_Sn phase (Ag_4_Sn: a = 2.9658 Å, c = 4.7842 Å; Ag_3_Sn: a = 2.9863 Å, c = 4.7840 Å) [34,37].

It should be noted that, to our knowledge, only the hexagonal variety has been reported for this solid solution (ζ). However, as for the Ag_3_Sn phase, the diffractogram cannot be explained by the existence of this allotropic variety alone due to the splitting of most of the peaks (except two).

We therefore hypothesized the existence of an orthorhombic variety for this phase, isostructural of that of the Ag_3_Sn phase. Because Ag_3_Sn and Ag_4_Sn exhibit the same structures at high temperature, the orthorhombic distortion of the lattice observed for Ag_3_Sn can reasonably occur for the ζ phase. Moreover, it was observed that a prolonged heating of the compound Ag_3_Sn at 320 °C leads to the formation of the solid solution ζ (Ag_4_Sn) [38]. It can therefore be assumed that this transformation is carried out by a mechanism of tin depletion concomitant with enrichment in Ag of the lattice of the Ag_3_Sn phase.

**Figure 6:**
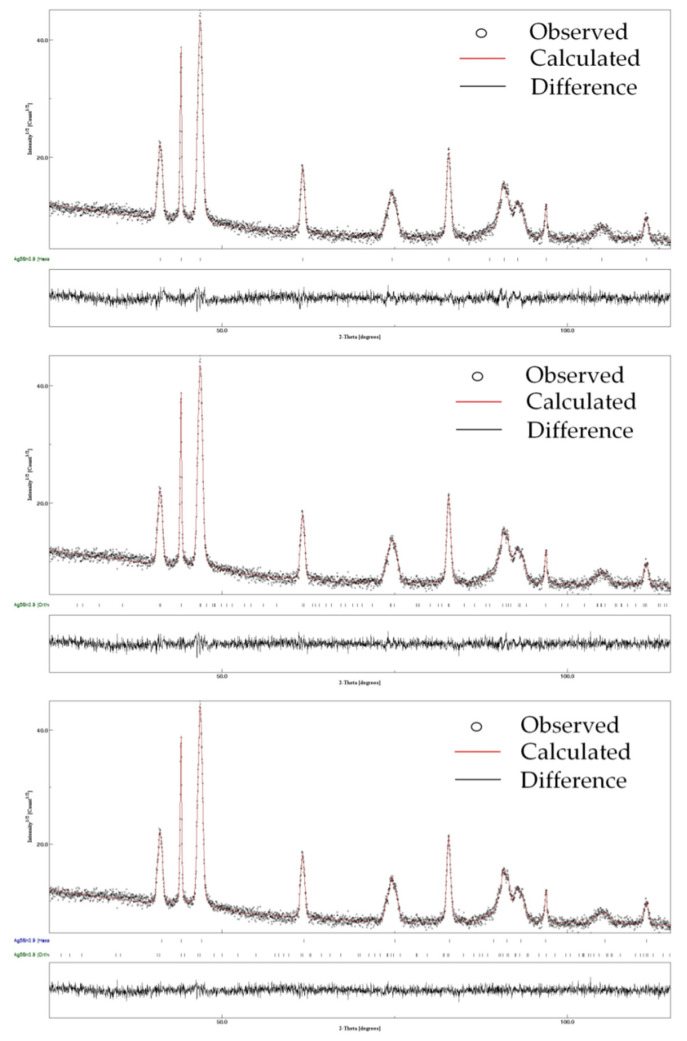
**Top**: Refinement based on the presence of only the hexagonal variety; **middle**: refinement based on the presence of only the orthorhombic variety; **bottom**: refinement based on the presence of the hexagonal variety and the orthorhombic variety.

Since tin totally occupies the 2a site (1/4 1/4 z = 0.1700) in the case of Ag_3_Sn (Pmmn group) [39], this mechanism allowing the transformation of Ag_3_Sn into Ag_4_Sn will likely occur via progressive substitution of Sn by Ag in the 2a site as shown in the Table 3. The occupancy rates of both elements of the 2a site are calculated based on EDX analysis so that the chemical formula of the obtained phase can be written Ag_3.4_Sn_0.6_ and corresponds to the compound obtained during this work: Ag_85_Sn_15_ (or Ag_5_Sn_0.9_). We therefore carried out refinements in the three hypotheses: (i) hexagonal variety alone, (ii) orthorhombic variety alone and (iii) presence of both varieties (Figure 6). The starting cell parameters and atomic positions were those given by King et al. for Ag_0.8_Sn_0.2_ [37] in the case of the hexagonal variety and those given by Fairhurst and al for the orthorhombic variety [39]. Thermal agitation (B_iso_) was fixed at 0.6 and not refined. In the orthorhombic Ag_5_Sn_0.9_ we imposed for the tin site (2a site) an occupancy rate equal to 0.6 for tin and occupancy rate equal to 0.4 for silver. The final atomic positions in hypothesis (iii) are reported in Table 3. Those for hypotheses (i) and (ii) are almost identical to those of hypothesis (iii) and therefore not shown. The refined diffractograms given in Figure 6 and Table 4 indicate the results of the refinements (cell parameters and reliability factors) in the three hypotheses.

As we can see, the refinement based on the presence of the two allotropic varieties leads to slightly better results (lower reliability factors and lower difference between calculated and observed XRD patterns as shown in the continuous background below the corresponding refinement). As shown for Ag_3_Sn, the synthesis in polyol medium leads to the formation of both allotropic varieties. However, it can be seen that high temperature variety remains predominant (59.9%) for the studied phase, whereas for Ag_3_Sn this allotropic variety represents the lower amount (11%).

## 4. Discussion

Jo et al. [40] used the polyol process to produce particles existing in the Ag–Sn system. In their protocol, the polyol (1,5-pentandediol) only plays the role of solvent allowing the synthesis to be carried out at 180 °C. Ag–Sn alloys are obtained via two steps: (i) obtaining Sn particles by the action of the NaBH_4_ reducing agent of the Sn^2+^ ions, and (ii) reduction of the Ag+ ions adsorbed on the Sn particles by the galvanic reaction (Equation (3)):

2Ag^+^ + Sn^0^ → Sn^2+^ + 2Ag^0^ (3)

Since no complementary analyses were used, authors concluded on the basis of X-ray diffraction, that the two phases (Ag_3_Sn and Ag_4_Sn) are present along with tin as impurity [40]. 

As for the synthesis of Ag_3_Sn [22], we took advantage of the reducing character of the polyol to carry out the synthesis by permuting the two steps described by Jo et al. First, metallic silver particles were obtained by reduction of Ag+ ions by simple action of the polyol. In a second step, the Sn^2+^ ions added to the medium were adsorbed on the surface of the silver particles. They then underwent a reduction thanks to the controlled addition of a strong reducing agent NaBH_4_. The formed tin atoms then diffused inside the silver particles to form submicron particles of the intermetallic compounds Ag_3_Sn [22] or ζ phase in the present study. It is worth noting that the main parameter governing the nature of the obtained phase is the amount of the reducing agent NaBH_4_. A high amount (NaBH_4_/Sn) of around 30 favors Ag_3_Sn formation whereas a lower amount (19.6) favors the phase rich in silver, namely ζ- Ag_5_Sn_0.9_.

During the investigation of the Ag–Sn system, appropriate analysis techniques such as DSC and EDX were necessary to qualify the formed phase (Ag_3_Sn or the ζ phase) (in our case the chemical formula is close to Ag_5_Sn). Indeed, the two phases have structural relationships leading to very similar diffractograms. Like us, several authors have reported similar diffractograms for samples qualified as Ag_3_Sn or the ζ phase (Ag_4_Sn) on the basis of EDX analysis [41,42].

We have proposed in this work a structural hypothesis to explain the similarity of the diffractograms observed for the two phases. It assumes that the Ag_3_Sn phase can evolve towards a phase richer in silver (ζ phase) thanks to a partial substitution of tin by silver on the 2a site of the Pmmn space group. This topotactic reaction is facilitated because the ζ phase and the high temperature variety of the Ag_3_Sn phase have the same symmetry and belong to the same space group (P6_3_/mmc) with very close cell parameters.

**Figure 7:**
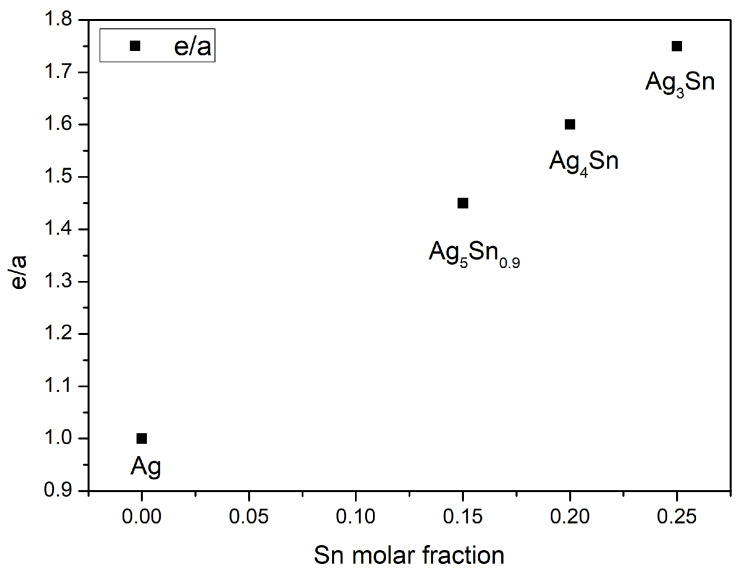
Evolution of electron concentration (e/a) as function of tin molar fraction.

Both Ag_3_Sn and ζ phases are classified as Hume-Rothery compounds [43]. Their structure and lattice parameters depend on the electron concentration per atom (e/a) [44]. To calculate the ratio e/a, silver involves one electron and tin involves four electrons. Figure 7 gives the evolution of (e/a) for the series of compounds existing in the Ag–Sn system. As can be seen, the concentration (e/a) increases with the molar fraction of tin present in the compound.

Table 5 compares the lattice parameters found during this work with those available in the literature for these compounds.

**Figure 8:**
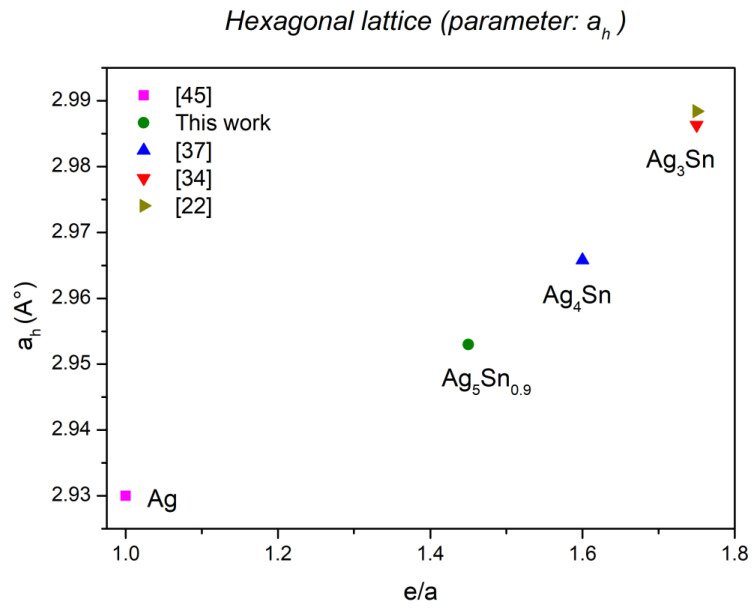
Evolution of the parameter a_h_ as function of (e/a).

**Figure 9:**
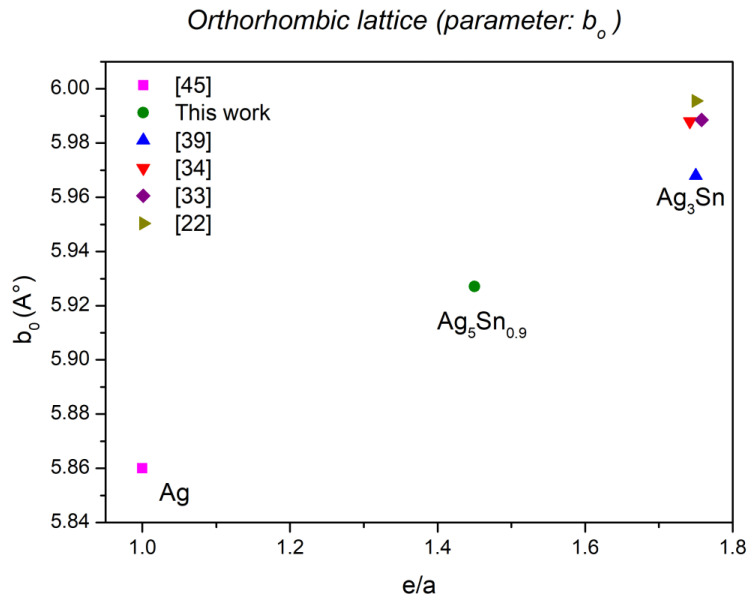
Evolution of the parameter b_o_ as function of (e/a).

It has been shown that for the phases of hexagonal symmetry, contrary to the parameter c_h_, the parameter a_h_ increases with the concentration (e/a) [43]. The lattice parameters determined for the Ag_3_Sn phase and the ζ phase (Ag_5_Sn_0.9_) are in good agreement with this correlation (Figure 8); they increase when the concentration (e/a) increases. Figure 9 represents the evolution of the orthorhombic lattice parameter b_0_ as a function of (e/a). This parameter actually derives from the a_h_ parameter as a result of the orthorhombic distortion (b_o_ ≈ 2a_h_). Similar to a_h_ for the hexagonal varieties, b_o_ also increases with the concentration (e/a).

For the ζ phase (Ag_5_Sn_0.9_), we hypothesized the existence of an orthorhombic symmetry as for Ag_3_Sn. The lattice parameter b_0_ obtained in this hypothesis is in good agreement with the predictions of Hume-Rothery [43]. In fact, its value correlates well with the concentration (e/a) for this chemical formula.

In addition, one notices that the e/a ratio has an influence on the compactness of the lattice. Indeed, c/a and the corresponding ratio in an orthorhombic system deviate from the hexagonal close packed (hcp) value, namely 1.633: it decreases when the electron density per atom (e/a) increases (see Table 5, column 7).

## 5. Conclusions

The Ag-Sn system presents an intermetallic compound of defined composition Ag_3_Sn with a non-congruent melting point at 480 °C and a solid solution called ζ phase extending over a restricted range of composition (9–16 at.% Sn) with a melting point that is also non-congruent but at a higher temperature (724 °C). This solid solution is also considered an intermetallic compound.

This work has been devoted to the synthesis of an intermetallic compound belonging to the ζ-phases by the polyol process. This ζ phase corresponds to the chemical formula Ag_5_Sn_0.9_. The particle’s size is close to 180 nm. The size of the crystallites is very close to that of the particles showing a monocrystalline character.

Despite different chemical compositions and melting temperatures, the two compounds exhibit substantially identical diffractograms. Ag_3_Sn exists in two allotropic varieties: one hexagonal existing at high temperature and the other with orthorhombic symmetry existing at low temperature. The two varieties have been the subject, in the literature, of structural studies showing a filiation between these two structures resulting from the orthorhombic distortion of the hexagonal symmetry with the relation b_0_ ≈ a_h_√3 (b_0_ being the parameter b of the orthorhombic cell). For the ζ phase, only a variety of hexagonal and isostructural with the formula Ag_3_Sn is reported in the literature (same space group and close lattice parameters). This variety does not allow a satisfactory explanation of the diffractogram observed for this phase. Pending a precise structural determination, it was necessary to admit the existence, for this phase, of an allotropic variety with orthorhombic and isostructural variants of the compound Ag_3_Sn. Taking into account this variety alongside the hexagonal variety leads to a better accounting of the results of the X-ray diffraction analysis. The lattice parameters obtained are in line with the Hume-Rothery rules.

## Figures and Tables

**Table 1:** Protocol of the synthesis of ζ-Ag_5_Sn_0.9_ particles in polyol medium.

Steps	Materials
Solvent	Ethylene Glycol
Surfactant (added at 50 °C)	PVP58000
Silver precursor and ratio (added at 70 °C)	AgNO_3_; m(PVP/AgNO_3_) = 1
t_160 °C_	5 min
Tin precursor and ratio	SnCl_2_; n(Ag/Sn) = 1.14
t_160 °C_	5 min
Reducing agent of tin	NaBH_4_; n (NaBH_4_/Sn) = 19.6With continuous mechanical stirring and controlled addition to 0.25 g/15 s
t_180 °C_	1 h
Cooling	Water quenching (≤5 min)
Centrifugation	12,000 tr/min
Drying of the powder	At 70 °C for 12 h

**Table 2:** Quantitative analysis of areas 1 and 2.

**Analysis of Spectrum: Spectra from Area 1**							
**Z**	**Element**	**Family**	**Atomic Fraction (%)**	**Atomic Error (%)**	**Mass Fraction (%)**	**Mass Error (%)**	**Fit Error (%)**
47	Ag	L	84.90	8.20	83.63	5.85	0.07
50	Sn	L	15.10	2.09	16.37	1.99	0.10
**Analysis of Spectrum: Spectra from Area 2**							
**Z**	**Element**	**Family**	**Atomic Fraction (%)**	**Atomic Error (%)**	**Mass Fraction (%)**	**Mass Error (%)**	**Fit Error (%)**
47	Ag	L	86.22	8.36	85.04	5.97	0.33
50	Sn	L	13.78	1.92	14.96	1.83	1.45

**Table 3:** Atomic positions in hypothesis (iii): presence of the two allotropic varieties.

Phase Variety	Atom	Quantity	Site	x	y	z	B_iso_	Occ
Orthorhombic and hexagonal varieties	Ag	4	4e	0.00477 (9)	1/4	0.66865 (5)	0.6	1
	Ag	2	2b	1/4	3/4	0.84618 (3)		1
	Sn	2	2a	1/4	1/4	0.18709 (3)		1 − x (0.6)
	Ag	2	2a	1/4	1/4	0.18705 (2)		x (= 0.4)
	Ag	2	2c	1/3	2/3	1/4		0.85
	Sn	2	2c	1/3	2/3	1/4		0.15

**Table 4:** Results of refinement of Ag_5_Sn_0.9_.

Hypothesis	Symmetry	Space Group	Cell Parameters (Å)	% Phase	Sigma	Rwp	Rexp
(i)	Hexagonal	P6_3_/mmc	a = 2.9550 (1) c = 4.7906 (1)	100	1.152	11.678	10.139
(ii)	Orthorhombic	Pmmn	a = 5.9182 (7) b = 4.7889 (1) c = 5.1612 (5)	100	1.133	11.481	10.127
(iii)	Hexagonal	P6_3_/mmc	a = 2.9530 (3) c = 4.7906 (2)	59.9	1.07	10.855	10.112
	Orthorhombic	Pmmn	a = 5.9271 (7) b = 4.7971 (4) c = 5.1823 (6)	40.1			

**Table 5:** Structural data of phases existing in the Ag-Sn system.

Phase (Symmetry)	Space Group	a (Å)	b (Å)	c (Å)	$\frac{2a}{\frac{c}{\sqrt{3}}+\frac{b}{2}}$	e/a	Reference
Ag_3_Sn (Orthorhombic)	Pmmn	4.78291 (2)	5.98854 (2)	5.15686 (2)	1.6019	1.75	[33]
	Pmmn	4.785 (1)	5.988 (1)	5.159 (1)	1.6023		[34]
	Pmmn	4.7802 (4)	5.968 (9)	5.1843 (9)	1.5995		[39]
	Pmmn	4.7882 (1)	6.0009 (2)	5.1699 (4)	1.5999		[22]
					$\frac{c}{a}$		
Ag_3_Sn (Hexagonal)	P6_3_/mmc	2.9863 (8)		4.7840 (2)	1.6019	1.75	[34]
	P6_3_/mmc	2.9755 (6)		4.7742 (7)	1.6045		[22]
					$\frac{c}{a}$		
Ag_4_Sn (Hexagonal)	P6_3_/mmc	2.9658		4.7824	1.6131	1.60	[37]
					$\frac{c}{a}$		
Ag_5_Sn_0.9_ (Hexagonal)	P6_3_/mmc	2.9530 (3)		4.7906 (5)	1.6222	1.45	This work
					$\frac{2a}{\frac{c}{\sqrt{3}}+\frac{b}{2}}$		
Ag_5_Sn_0.9_ (Orthorhombic)	Pmmn	4.7971 (4)	5.9271 (7)	5.1823 (6)	1.6109	1.45	This work
					$\frac{c}{a}$		
Ag (Hexagonal)	P6_3_/mmc	2.93 (1)		4.79 (1)	1.633	1.00	[45]

## Data Availability

Part of the data presented in this study is available within the article (MET + EDX) and the other part (XRD and DTA) is presented at https://hal.archives-ouvertes.fr/hal-03861729.
